# *HOTAIR*: A Promising Long Non-coding RNA with Potential Role in Breast Invasive Carcinoma

**DOI:** 10.3389/fgene.2017.00170

**Published:** 2017-11-21

**Authors:** Niloofar Avazpour, Mohammadreza Hajjari, Maryam Tahmasebi Birgani

**Affiliations:** ^1^Department of Genetics, Faculty of Science, Shahid Chamran University of Ahvaz, Ahvaz, Iran; ^2^Department of Medical Genetics, School of Medicine, Ahvaz Jundishapur University of Medical Sciences, Ahvaz, Iran

**Keywords:** HOTAIR, long non-coding RNA, gene expression, cancer, breast invasive carcinoma

The majority of the Human genome is transcribed into a large number of non-coding RNAs (ncRNAs) which have different roles in the cell (Haemmerle and Gutschner, 2015). Long non-coding RNAs (LncRNAs) constitute a heterogeneous group of the ncRNAs that are longer than 200 nt. They are usually capped and poly-adenylated like mRNAs (Esteller, 2011; Fritah et al., 2014). Accumulating evidence show that the lncRNAs play an important role in cancer progression. So, these molecules have been considered as potential biomarker and therapeutic targets (Hajjari and Khoshnevisan, 2013; Hajjari et al., 2013b; Huarte, 2015).

One of the well-known lncRNAs is *HOTAIR (HOX transcript antisense RNA)* which is known to effect on the chromatin structure (Cao, 2014; Huang et al., 2014). This trans-acting lncRNA has different target loci including tumor suppressor genes. It recruits the PRC2 and LSD1 complexes in order to repress the transcription of target genes (Hajjari and Salavaty, 2015).

Owing to this regulatory mechanism, lots of the studies reported the role of *HOTAIR* in progression of different cancers such as breast, colon, and gastric cancer (Reviewed in Hajjari et al., 2013a, 2014; Hajjari and Salavaty, 2015). Gupta et al. found the dysregulation of this long transcript in breast tumors (Gupta et al., 2010). Then, other studies showed the importance of the *HOTAIR* in poor prognosis, metastasis, invasion, and short overall survival of breast cancer. Alves et al. indicated the potential role of *HOTAIR* in EMT progression and cancer stem cell features (Pádua Alves et al., 2013). Also, Gökmen-Polar et al. showed the *HOTAIR* as a marker for lymphatic metastases in ER-negative patients (Gökmen-Polar et al., 2015). However, the results are in contrast to the results of Sorensen et al but are similar to those reported by Lu et al. (Sørensen et al., 2013; Liu et al., 2014).

There are just a few studies with limited number of samples indicating the differentiated expression of *HOTAIR* in breast tumors compared to normal tissues. We believe that the differential expression of *HOTAIR* may indicate the potential role of this lncRNA in cancer initiation and progression. This study was aimed to highlight the potential role of *HOTAIR* in breast cancer. In this study, we analyzed the *HOTAIR* expression in breast invasive carcinoma tissues derived from TCGA (The Cancer Genome Atlas) which applies RNA sequencing of large cohorts. We also validated our data in different GEO dataset. The results can highlight the role of *HOTAIR* in breast invasive cancer and provide the viewpoint for further analyses of *HOTAIR* in breast cancer progression.

For this study, The Cancer Genome browser database (https://genome-cancer.ucsc.edu/), which uses the TCGA data, was used to analyze the association between *HOTAIR* expression level and features of breast invasive carcinoma. “BRCA gene expression (Illumina Hiseq Percentile)” data was selected for our analysis. The expression of *HOTAIR* was compared between the tumor (*n* = 1,066) and normal samples (*n* = 133) of TCGA tissues. Besides, the association between *HOTAIR* expression level of breast tissues and ER/PR/HER2 status of tumor tissues was also checked.

For validation study, the expression of *HOTAIR* gene was analyzed between breast tumor and normal tissues in different GEO datasets and profiles including GSE58135, GDS2618, GDS3853, GSE69240, GSE48408 through NCBI as well as Nexus expression database (http://syslab4.nchu.edu.tw/).

Normalized expression (Z score) derived from TCGA tissues of cancer genome browser were compared between groups of study. The statistical analysis was done by *t*-test through R-software integrated in cancer genome browser. The *P*-value <0.05 were considered as significant *P*-value. Expression analysis between breast invasive tumor and normal tissues showed that the *HOTAIR* is significantly overexpressed in breast tumors compared to normal tissues (Figure 1). Categorizing the samples based on the HER2/PR/ER status demonstrated that *HOTAIR* is significantly over expressed in HER2 positive samples compared to negative ones. Additionally, in comparison with ER and PR positive tumors, *HOTAIR* is up-regulated in ER and PR negative tumors. It was found that the expression of *HOTAIR* is down-regulated in LuminalA, LuminalB, and normal like breast tumors subtypes (Data not shown).

**Figure 1:**
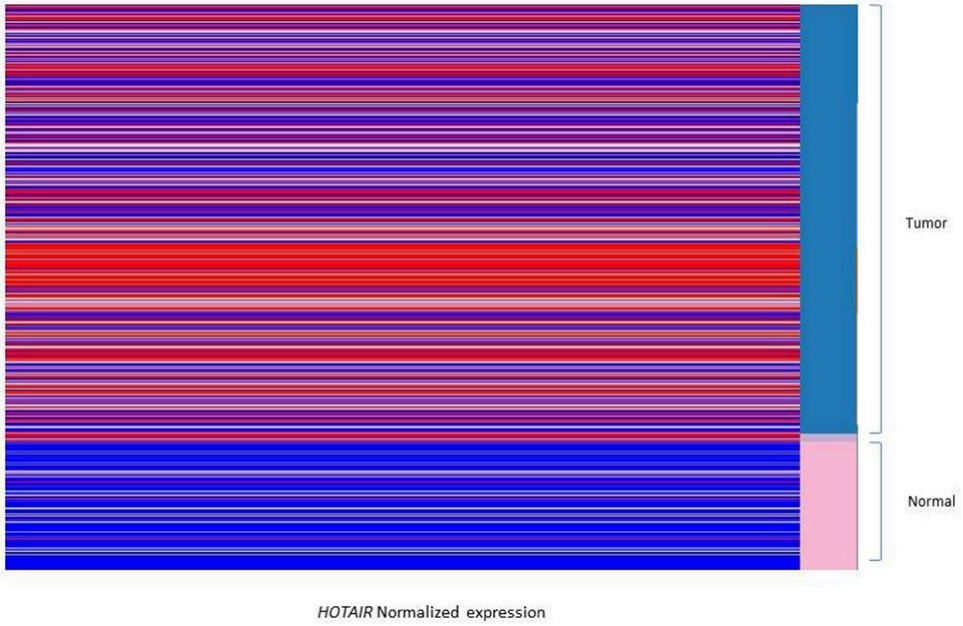
Heatmap displays the comparison between the *HOTAIR* expression of breast tumors and solid normal tissues. The data heatmap displays the normalized gene expression in red (Z > 0) and blue color (Z < 0). The *HOTAIR* is overexpressed in breast tumor tissues (A part of the tissues are shown as blue subgroup in right column) compared to normal tissues (shown as pink subgroup in right column). Data is derived from Cancer genome browser database.

Different GEO dataset and profiles were analyzed in order to compare the *HOTAIR* expression level between tumor and normal samples. The expression level between tumor and normal tissues were compared by *t*-test through GraphPad software. The data showed that the *HOTAIR* is significantly up-regulated in cancer cell lines and tumor tissues compared to normal breast samples (Table 1). To our knowledge, this is the first integrative study highlighting the over expression of *HOTAIR* in breast invasive carcinoma in a large cohort and different data sets. Since the breast cancer is a heterogeneous disease, the predictive power of current biomarkers is sometimes limited. So, there is a need to identify additional prognostic and predictive molecular biomarkers. The aim of this study was to examine the significance of the *HOTAIR* gene expression in breast cancer. Given the importance of *HOTAIR* in breast cancer, it promises as a potential biomarker and therapeutic target. However, because of the follow up limitations of TCGA cohort, further studies are necessary to reveal the role of *HOTAIR* in breast cancer initiation/progression in different cohorts with well annotation for tumor histology, and survival data.

**Table 1:** The Up-regulation of HOTAIR in breast cancer samples (case) compared to normal samples (controls) in different GEO datasets.

**Dataset**	**Case (N)**	**Control (N)**	***P*-value**
GSE58135	Triple negative Breast cancer (42)	Adjacent breast tissues (21)	8.75E-8
	Breast cancer cell lines (28)	Adjacent breast tissues (21)	5.78E-6
	ER+/HER2- Breast cancer primary tumors (42)	Adjacent breast tissues (21)	2.94E-6
GDS2618	Cancer cell lines (9)	Normal breast cell line (3)	0.00024
GDS3853	Ductal carcinoma (14)	Healthy breast (5)	<0.0001
GSE69240	High grade ductal carcinoma in situ (25)	Normal breast organoids (10)	1.92E-8
GSE48408	High metastasis (82)	Low metastasis (82)	0.0076

## Author contributions

All authors listed have made a substantial, direct and intellectual contribution to the work, and approved it for publication.

### Conflict of interest statement

The authors declare that the research was conducted in the absence of any commercial or financial relationships that could be construed as a potential conflict of interest.
